# Rare and potential pathogenic mutations of *LMNA* and *LAMA4* associated with familial arrhythmogenic right ventricular cardiomyopathy/dysplasia with right ventricular heart failure, cerebral thromboembolism and hereditary electrocardiogram abnormality

**DOI:** 10.1186/s13023-022-02348-z

**Published:** 2022-05-07

**Authors:** Jia Chen, Yuting Ma, Hong Li, Zhuo Lin, Zhe Yang, Qin Zhang, Feng Wang, Yanping Lin, Zebing Ye, Yubi Lin

**Affiliations:** 1 The First Dongguan Affiliated Hospital, Guangdong Medical University, Dongguan, 523710 Guangdong Province China; 2 The Second Department of Cardiology, Department of Obstetrics and Gynecology, The Second People’s Hospital of Guangdong Province, Guangzhou, 510310 China; 3 College of Life Sciences, University of Chinese Academy of Sciences, Beijing, 100049 China; 4 School of Public Health, Jinzhou Medical University, Jinzhou, 121001 China; 5 The Center of Cardiovascular Diseases, The Department of Cardiology, The Fifth Affiliated Hospital, Sun Yat-Sen University, Zhuhai, 519000 China; 6 Guangdong Provincial People’s Hospital, Guangdong Academy of Medical Sciences, Guangdong Geriatrics Institute, Guangdong Cardiovascular Institute, Guangzhou, 510080 China

**Keywords:** Cardiomyopathy, Arrhythmogenic right ventricular cardiomyopathy/dysplasia, Sudden death, Arrhythmia, Thromboembolism, Gene

## Abstract

**Background:**

Arrhythmogenic right ventricular cardiomyopathy/dysplasia (ARVC/D) is associated with ventricular arrhythmia, heart failure (HF), and sudden death. Thromboembolism is also an important and serious complication of ARVC/D. However, the etiology of ARVC/D and thromboembolism and their association with genetic mutations are unclear.

**Methods:**

Genomic DNA samples of peripheral blood were conducted for whole-exome sequencing (WES) and Sanger sequencing in the ARVC/D family. Then, we performed bioinformatics analysis for genes susceptible to cardiomyopathies and arrhythmias. Further, we analyzed how the potential pathogenic mutations were affecting the hydrophobicity and phosphorylation of amino acids and their joint pathogenicity by ProtScale, NetPhos and ORVAL algorisms.

**Results:**

We discovered a Chinese Han family of ARVC/D with right ventricular HF (RVHF), cerebral thromboembolism, arrhythmias (atrial fibrillation, atrial standstill, multifocal ventricular premature, complete right bundle block and third-degree atrioventricular block) and sudden death. Based on the WES data, the variants of *LMNA* p.A242V, *LAMA4* p.A225P and *RYR2* p.T858M are highly conserved and predicated as “deleterious” by SIFT and MetaSVM algorithms. Their CADD predicting scores are 33, 27.4 and 25.8, respectively. These variants increase the hydrophobicity of their corresponding amino acid residues and their nearby sequences by 0.378, 0.266 and 0.289, respectively. The *LAMA4* and *RYR2* variants lead to changes in protein phosphorylation at or near their corresponding amino acid sites. There were high risks of joint pathogenicity for cardiomyopathy among these three variants. Cosegregation analysis indicated that *LMNA* p.A242V might be an important risk factor for ARVC/D, electrocardiogram abnormality and cerebral thromboembolism, while *LAMA4* p.A225P may be a pathogenic etiology of ARVC/D and hereditary electrocardiogram abnormality.

**Conclusions:**

The *LMNA* p.A242V may participate in the pathogenesis of familial ARVC/D with RVHF and cerebral thromboembolism, while *LAMA4* p.A225P may be associated with ARVC/D and hereditary electrocardiogram abnormality.

## What is new?

1. We first demonstrated that *LMNA* p.A242V might participate in the pathogenesis of familial ARVC/D with right ventricular heart failure (RVHF) and cerebral thromboembolism, while *LAMA4* p.A225P may be associated with ARVC/D and hereditary electrocardiogram abnormality.
2. The phenotypes of LMNA p.A242V/LAMA4 p.A225P presented with ARVC/D and complex complications, including RVHF, atrial fibrillation, atrial standstill, multifocal ventricular premature, right bundle block and third-degree atrioventricular block, which were the high-risk factors for thromboembolism and sudden death.
3. Anticoagulant therapy may need to be considered for patients with ARVC/D induced by LMNA p.A242V.

## Introduction

Arrhythmogenic right ventricular cardiomyopathy/dysplasia (ARVC/D) is characterized by progressive lipid storage and fibro-fatty replacement of normal cardiomyocytes in both ventricles. It is associated with ventricular arrhythmia, heart failure (HF), and sudden cardiac death (SCD), especially in young people [1–3]. ARVC/D commonly occurred with early precordial QRS fragmentation and malignant ventricular tachycardia of hemodynamic compromise [2, 4]. Some patients with implantable cardioverter-defibrillator (ICD) still complicate SCD induced by repeated electrical storms. Thromboembolism, especially pulmonary thromboembolism, is an important and serious complication of ARVC/D [5–9]. In our previous report [9], the patient with ARVC/D induced by *DSG2* p.F531C mutation occurred with cerebral thromboembolism. Patients with ARVC/D and thrombus formation are at higher risk of a poor outcome and even caused sudden death induced by pulmonary thromboembolism [10]. Anticoagulation should be used in ARVC/D patients with large, hypokinetic right ventricle and slow blood flow [7]. This study found a Chinese Han family with ARVC/D, complicating bilateral low-limb edema caused by right ventricular heart failure (RVHF), complex arrhythmias, cerebral thromboembolism, hereditary electrocardiogram (ECG) abnormality and unexplained sudden death. In this ARVC/D family, four members presented with RVHF. Two of them occurred with severe cerebral thromboembolism, resulting in long-term bed rest, and one of them had sudden death at night. The etiology of ARVC/D and cerebral thromboembolism and whether thromboembolism is related to ARVC/D are unclear. Multiple genes, including desmoplakin, plakophilin-2, desmoglein-2, desmocollin-2, transforming growth factor-β3, ryanodine receptor-2 (*RYR2*), and transmembrane protein-43, plakoglobin, desmin, titin, phospholamban lamin A/C (*LMNA*), and catenin-α-3 have been identified in ARVC/D patients [11, 12]. Whole-exome sequencing (WES) combined with risk genes filter and cosegregation analysis is used to detect the possible disease-causing mutation [13]. We would further explore whether this familial ARVC/D and complex complications, especially thromboembolism, were associated with genetic background in this study.

## Materials

### Ethical compliance

All the participants have signed the informed consent. All procedures performed in the study involving human participants followed the Declaration of Helsinki and ethical standards approved by the Guangdong Medical Institutional Review Board and Medical Ethics Committees [No.GDREC2016001H (R1)]. Detailed clinical information was collected. The clinical information included family history, age of presentation, initial symptoms of arrhythmia, physical examination, electrocardiogram (ECG), echocardiogram and cardiovascular magnetic resonance (CMR) based on their informed consent. The current study identified an ARVC/D family using the 2010 ARVC/D Revised Task Force Criteria [14].

### Clinical presentation and familial characteristics

**Fig. 1:**
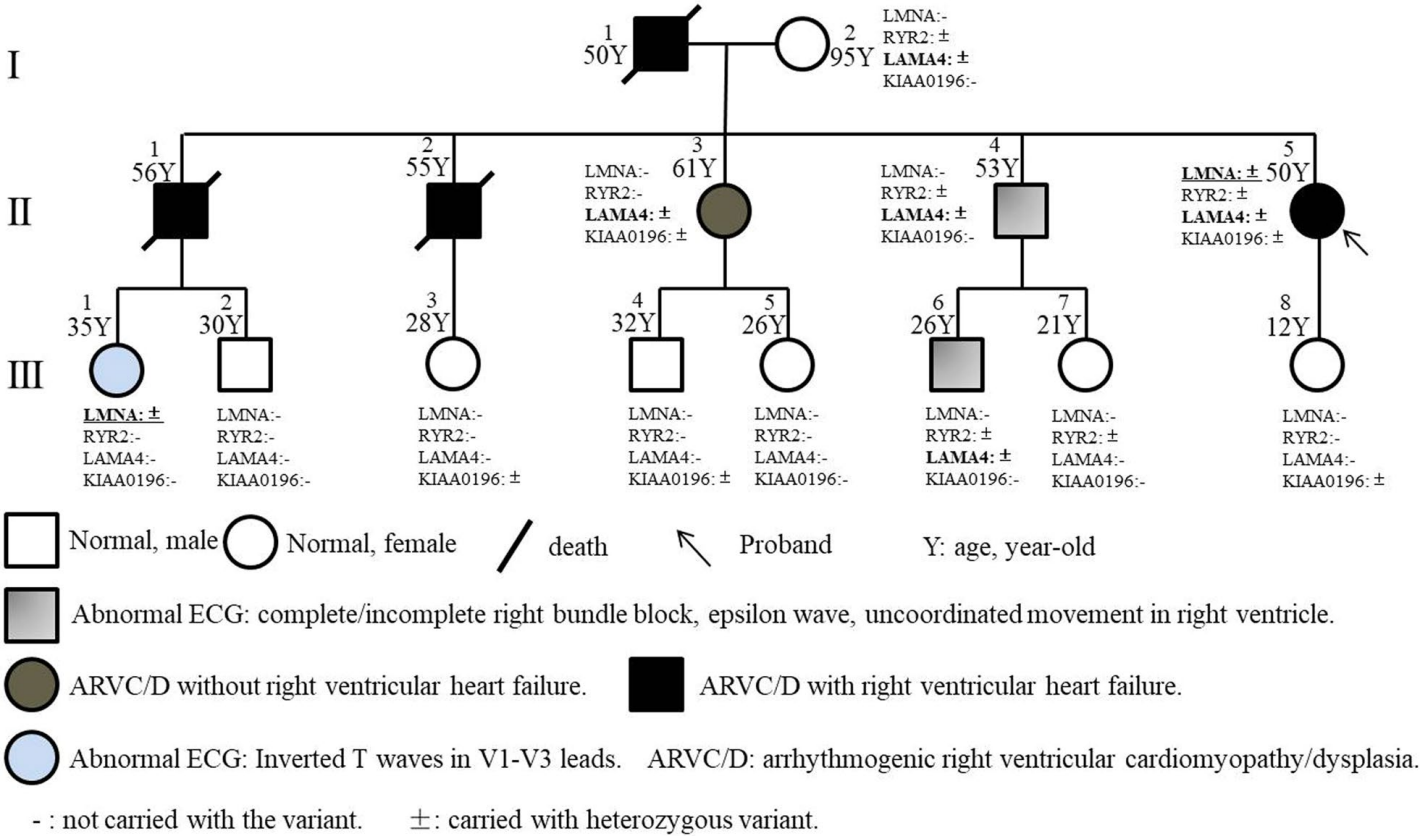
Familial pedigree

We discovered a Chinese Han family of ARVC/D (Fig. 1) with familial history of RVHF, cerebral thromboembolism, arrhythmias and unexplained sudden death. The patient of II: 4 (male, 53-year old) went to our hereditary arrhythmia clinic for genetic counseling due to a family history of cardiac edema and a complete right bundle block. He suffered from progressive right bundle block without clinical symptoms. His sister, the patient of II: 5 (female, 50-year old), occurred from atrial fibrillation for ten years and was orally administrated with Metoprolol. She felt shortness of breath when climbing two floors and appeared with severe edema of both lower extremities due to RVHF since 2016. The diagnosis of echocardiography and CMR was accordant with the primary and secondary criteria of ARVC/D. The B-type natriuretic peptide precursor of II: 5 was slightly elevated by 308.9 pg/ml. Similar to II: 5 patient, II: 1 (male, 56-year old) and II: 2 (male, 55-year old) patients also repeat complicated with obvious RVHF and severe edema of both lower extremities. The members of I: 1 (male) and II: 1 died of severer edema of low limbs induced by RVHF and cerebral ischemia stroke at the age of 50 and 56-year-old, respectively, while II: 2 occurred with severer edema of low-limbs due to RVHF and subsequently died of unexplained sudden death when sleeping at the age of 55-year old. The etiology of cerebral thromboembolism of 1: 1 and II: 1 didn’t exclude cardiogenic thromboembolism, especially when the ARVC/D pathogenesis was involved in the left ventricle. II: 4 and the third-generation family members have no arrhythmia event or clinical symptoms.

### The electrocardiogram characteristics

**Fig. 2:**
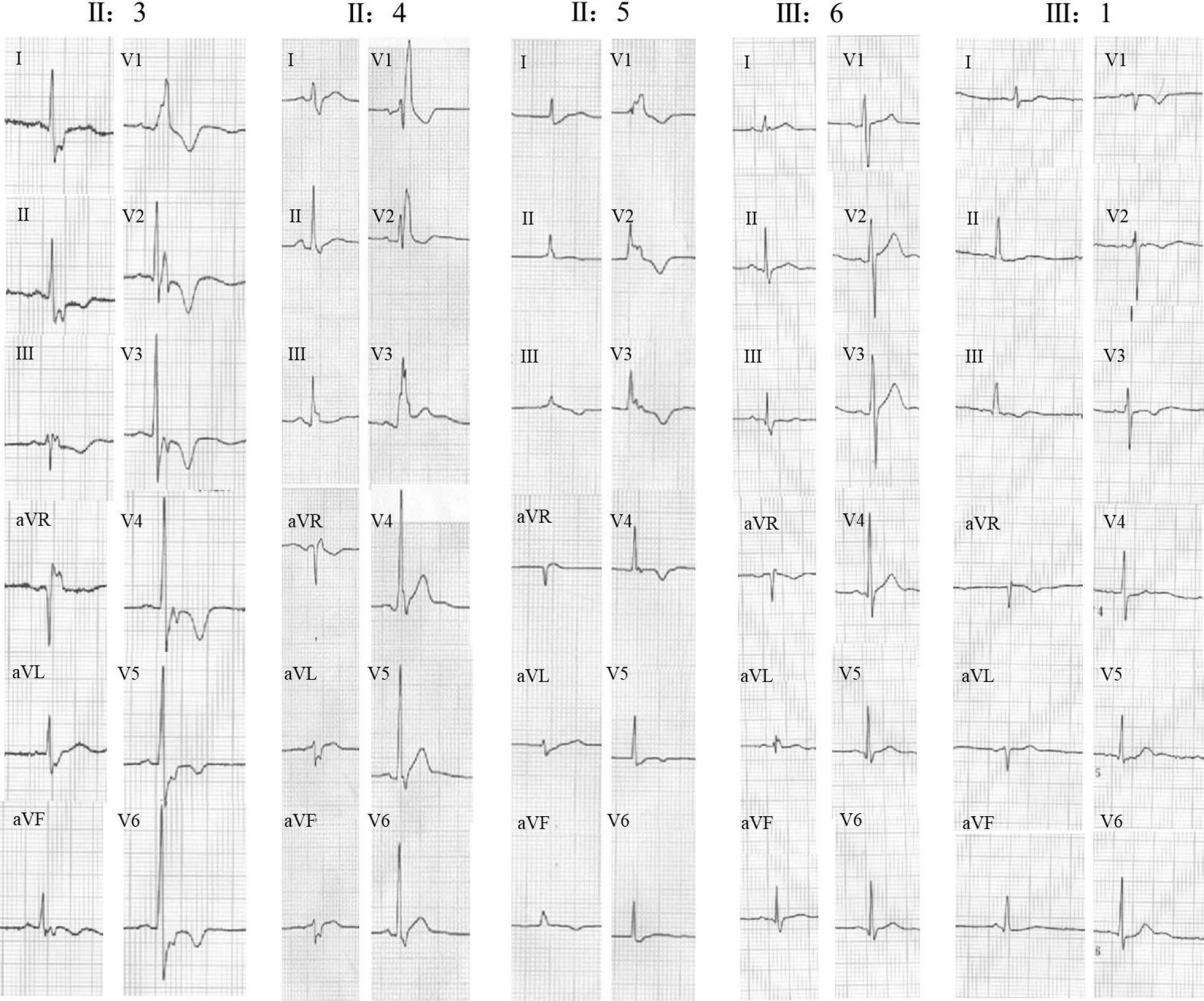
The electrocardiogram characteristics

The ECG characteristics of II: 5 present with atrial fibrillation, atrial standstill, low voltage of limb leads, multifocal ventricular premature, complete right bundle block, epsilon waves in V_2_-V_3_ lead and inverted T waves in 2016 (Fig. 2 and Table 1). The patient of II: 5 was implanted with a permanent pacemaker in another hospital due to repeat syncope induced by the third-degree atrioventricular block in 2021. The ECG of II: 3 shows the complete right bundle block, epsilon waves in V_2_-V_4_ lead and inverted T waves, while the ECG of II: 4 presents the complete right bundle block. The ECG of III: 6 indicates the early change of incomplete right bundle block, characterized by enlarging S waves in II, III, aVF, and V_5_-V_6_ leads, concomitant with abnormal q waves in III, aVF and V_4_-V_6_ leads, also flat T waves in III, V_5_-V_6_ leads and abnormal positive T wave in V_1_ lead. The abnormal voltage of T wave in V_1_ lead was higher than that of T waves in V_5_-V_6_ lead. The ECG of III: 1 shows the fat and inverted T waves in II, III, aVF and V_1_-V_4_ lead.

**Table 1:** The characteristics of CMR and ECG

Variables	II:3	II:4	II:5	III:6
Age (years)	61	53	50	26
Sex	F	M	F	M
LVEF (%)	72.64	54.66	63.48	61.33
LVEDVI (mL/m^2^)	77.44	80.56	84.39	72.95
LVESVI (ml/m^2^)	21.20	36.52	30.82	28.21
LVCO (L/min)	4.93	5.10	3.59	6.35
LV involvement	+	−	−	−
LV fatty infiltration	−	−	+	−
LV LGE	−	−	+	−
LV global abnormality	−	−	−	−
RVEF (%)	57.10	48.48	36.39	50.30
RVEDVI (mL/m^2^)	72.85	89.43	151.10	71.25
RVESVI (mL/m^2^)	31.25	46.08	21.68	35.41
RVCO (L/min)	3.64	4.80	3.68	5.09
RV global abnormality	+	−	+	−
RV fatty infiltration	+	−	+	−
RV LGE	−	−	−	−
Abnormal q waves in II, III and aVF	−	−	−	+
Complete right bundle branch brock	+	+	+	−
T wave inversion	+	−	+	−
S wave delay	+	+	+	+
Epsilon wave in V_1_–V_3_	+	−	+	−
Ventricular arrhythmia	+	+	−	−
Atrial fibrillation	−	−	+	−

LVEF: left ventricular ejective fraction. LVEDVI: left ventricular end-diastolic volume index. LVESVI: left ventricular end-systolic volume index. LVCO: left ventricular cardiac output. LV: left ventricular/ventricle. RV: right ventricular/ventricle. LGE: late gadolinium enhancement. CMR: cardiovascular magnetic resonance. RVEF: right ventricular ejective fraction. RVEDVI: right ventricular end-diastolic volume index. RVESVI: right ventricular end-systolic volume index. ECG: electrocardiogram. −: there was no such change

### The characteristics of cardiovascular magnetic resonance and echocardiogram

**Fig. 3:**
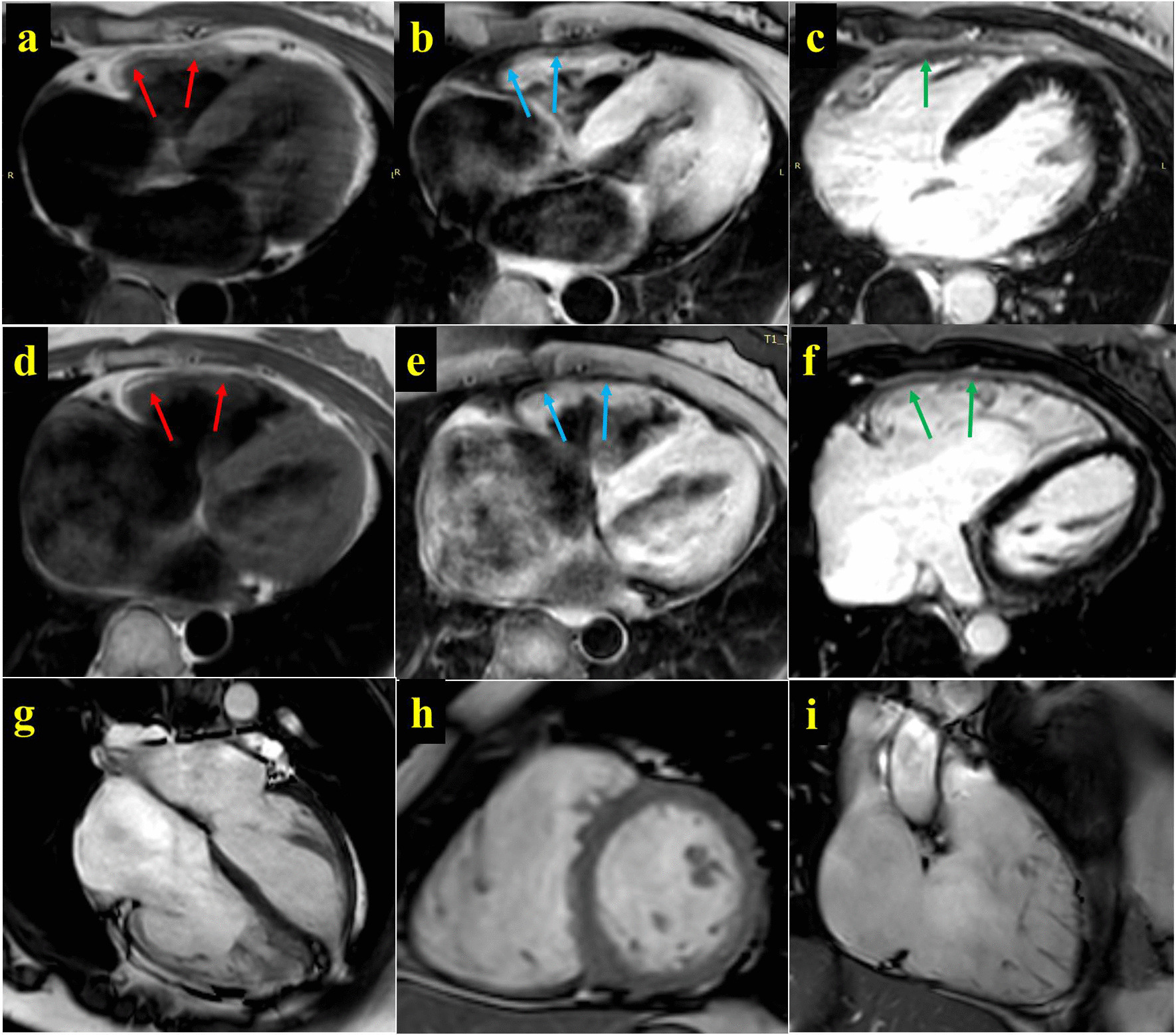
The characteristics of cardiovascular magnetic resonance. The CMR of II: 3. **a**–**c** shows the delayed enhancement scanning PSIR images of T1W, T1W-FS and Gd-DTPA on the body axis. These images indicate the thickening of the free wall of the right ventricle with fat signal (**a** T1W, red arrow, and high signal; **b** lipid pressure of T1W, blue arrow, and low signal). Gd-DTPA delayed enhancement scanning shows mild local enhancement, suggesting the change of myocardial fibrosis. The CMR of II: 5. **d**–**f** shows the delayed enhancement scanning PSIR images of T1W, T1W-FS and Gd-DTPA on the body axis. These images indicate the thickening of the free wall of the right ventricle with fat signal (**a** T1W, red arrow, and high signal; **b** lipid pressure of T1W, blue arrow, and low signal). Figure **g**–**i** are the diastolic images of B-TFE film sequence for four-chamber heart, short-axis position and two-chamber heart of right ventricle, showing the obvious expansion of right atrium and right ventricular cavity

The CMR characteristics and echocardiogram (Fig. 3 and Table 1) of II: 5 showed obvious fat infiltration in the right ventricular free wall, decreased right ventricular wall motion, increased right ventricular volume and decreased overall systolic function, which met the main diagnostic criteria of ARVC/D. The free wall of the basal segment in the left ventricle had pericardial fat infiltration, while the left ventricular volume and the overall systolic movement were normal. There was also moderate tricuspid regurgitation.

The CMR and echocardiogram of II: 3 indicated remarkable fat infiltration and uncoordinated movement in the right ventricular free wall, while the right ventricular systolic function was normal. Additionally, the left ventricular apex was relatively hypertrophic, and the left ventricle was slightly enlarged, although the left ventricular systolic function was normal.

The CMR of III: 6 showed normal cardiac structure and function. The echocardiograms of II: 4 and III: 6 illustrated that the middle-lower segment of the free wall in the right ventricle became thin, with some parts bulging outward like shallow sacs. The thinnest segment was in the right ventricular apex with 2.1 and 1.6 mm for II: 4 and III: 6, respectively, which also accompanied the disordered arrangement of muscular trabeculae. The echocardiogram of III: 1 showed normal cardiac structure and function by echocardiogram.

## Methods

### Whole exome sequencing

We extracted her blood for WES because the II: 5 proband presented a typical ARVC/D phenotype and complex complications. Genomic DNA samples of II: 5 were isolated from peripheral blood using a standard DNA extraction protocol. The isolated genomic DNA was then fragmented into 150–200 bp and subjected to DNA library preparation using established Illumina paired-end protocols. Adaptor-ligated libraries were amplified via PCR. A portion of each library was used to create an equimolar pool. Each pool was amplified to enrich targets sequenced by the Agilent SureSelectXT Target Enrichment System (Agilent Technologies Inc., Santa Clara, CA, USA). According to the manufacturer's protocol, whole-exome capture was performed with the Agilent SureSelectXT Human All Exon 50 Mb Kit (Agilent Technologies Inc.). According to the manufacturer's instructions, the exome-enriched libraries were sequenced with the Illumina Hiseq 2000 platform (Illumina, San Diego, CA, USA), and 100 bp paired-end sequencing reads were generated. Each sample was sequenced per lane to obtain an average theoretical depth of 100×.

### Read mapping, variant detection, and functional annotation

After WES, raw reads were collected for quality control, in which low-quality reads were filtered, and 3′/5′ adapters were trimmed using the Trim Galore program. Clean reads were aligned to the human reference genome (University of California Santa Cruz, UCSC build hg19) using the Burrows-Wheeler Aligner (BWA) program. The quality scores were recalibrated, and reads were realigned to the reference genome using the Genome Analysis Toolkit (GATK) software package. Following the exclusion of duplicate reads, insertion-deletions (InDels) and single-nucleotide polymorphisms (SNPs) were called using the GATK or Sequence Alignment/Map tools (SAM tools).

### Pathogenic risk classification

For the data from WES of II: 5 proband, SNPs and Indels were annotated using a pipeline, in which all insertion and deletion variants occurring at coding regions were considered damaging, and nonsynonymous SNPs were predicted by SIFT (http://sift.jcvi.org/www/), PolyPhen-2 (Polymorphism Phenotyping v2, http://genetics.bwh.harvard.edu/pph2/) and MetaSVM [15]. Subsequently, the common risk genes associated with cardiomyopathies and arrhythmias, as reported in our previous research [16], were detected for the II: 5 proband. These variants were screened, and the filtering criteria were as follows: (1) same variants in the WES data; (2) missense, nonsense, insertion and deletion variants; (3) SNPs with minor allele frequency, not ≥ 0.01 according to the SNP database of National Center; excluded variants with allele frequency in gnomAD (all) higher than 1%, or higher than 5% in house frequency. The potential risk variants were classified as “pathogenic (P)”, “likely pathogenic (LP)”, “uncertain significance (US/VUS)”, “likely benign (LB)” or “benign (B)” by the Clinvar database and InterVar tool [17] following the 2015 ACMG/ACP guidelines [18]. The detailed ACMG classification was shown in our previous research [16].

The variants were also excluded if the CADD predicting score was below 20. The higher CADD scores indicate that a variant is more likely to have harmful effects. A scaled score of 10 or greater indicates a raw score in the top 10% of all possible reference genome SNVs, and a score of 20 or greater indicates a raw score in the top 1%. Synonyms variants and those with a CADD predicting score lower than 20 were excluded unless reported as pathogenic or likely pathogenic in ClinVar [19].

### Conservation analyses of the risk variants

To determine whether the variants, including *LMNA*, *LAMA4* and *RYR2* genes, were conserved in the species and assess their possible pathogenicity, we searched for candidate proteins from the universal protein knowledgebase (UniProt)[20]. Multiple cross-species amino acid sequences were selected in the list and extracted into FASTA format. For Multiple Sequence Alignment, T-Coffee (https://www.ebi.ac.uk/Tools/msa/tcoffee/) was used to compare the conservation of target variant sites.

### Sanger sequencing in the family members

Genomic DNA samples of the family members were isolated from peripheral blood using a standard DNA extraction protocol. When the suspected pathogenic variants were obtained in each step, they were screened again using Sanger sequencing in the other family members, aiming to conduct a subsequent cosegregation analysis of genotypes and phenotypes among family members. The primers were designed by Primer Premier 5.0 and are shown in Table 2. The method of Sanger sequencing has been detailed in our previous study [11, 21]

**Table 2:** The primers for the Sanger sequence

Genes	Variant detection	Forward primer	Backward primer
*LMNA*	Chr1:156104681	5′AATTCTGATTTTGGTTTCTGT3′	5′GGGTTAGGACTGTGGTGAC3′
*ACTA1*	chr1:229568310	5′GTCGGTATGGGTCAGAAAGAT3′	5′ATGAAGGAGGGCTGGAAGA3′
*RYR2*	chr1:237666765	5′TGACCAGTTATTACGGACAT3′	5′ATACCAGTGATTGCCATTC3′
*MYL2*	chr12:111356969	5′TTGGAGACCTGAGTGTGGA3′	5′TGGGATTGTTTGGAGGATAG3′
*MYH7*	chr14:23902749	5′GAAGGGACTCACTGGTAACTC3′	5′CTCTTTGGAGGGTCTGGA3′
*TPM1*	chr15:63336266	5′CCTGTCTTTCCCTCTGTCTCT3′	5′AGTCCAATTTCAGCTGCTCTAT3′
*TTN*	chr2:179414991	5′ACCCCATAGTCCAAATTCTCA3′	5′AGTCTGCTGGTTTCCCGTT3′
*TTN*	chr2:179426798	5′GTGAACTGGAGAAAAGATGGTC3′	5′CTGGGGGACTGAATGGATA3′
*TTN*	chr2:179435192	5′CCTGTTCAATACTTTCACTGT3′	5′GCTGGTTTCTCTCCTTTC3′
*TTN*	chr2:179435225	5′CCTGTTCAATACTTTCACTGT3′	5′GCTGGTTTCTCTCCTTTC3′
*TTN*	chr2:179435281	5′CCTGTTCAATACTTTCACTGT3′	5′GCTGGTTTCTCTCCTTTC3′
*TTN*	chr2:179435636	5′CCTGTTCAATACTTTCACTGT3′	5′GCTGGTTTCTCTCCTTTC3′
*TTN*	chr2:179435664	5′CCTGTTCAATACTTTCACTGT3′	5′GCTGGTTTCTCTCCTTTC3′
*TTN*	chr2:179435678	5′CCTGTTCAATACTTTCACTGT3′	5′GCTGGTTTCTCTCCTTTC3′
*TTN*	chr2:179436004	5′CCTGTTCAATACTTTCACTGT3′	5′GCTGGTTTCTCTCCTTTC3′
*TTN*	chr2:179436043	5′CCTGTTCAATACTTTCACTGT3′	5′GCTGGTTTCTCTCCTTTC3′
*TTN*	chr2:179436062	5′CCTGTTCAATACTTTCACTGT3′	5′GCTGGTTTCTCTCCTTTC3′
*TTN*	chr2:179439808	5′AGCCAACAGAAACTACAGAGCC3′	5′GGTCCCAAGAGAAGGTTACAAA3′
*TTN*	chr2:179440715	5′GAGCATCAAAAAGTAGGAGAC3′	5′GTGGTGGCTGTGGAATAA3′
*TTN*	chr2:179442904	5′TTCACCAAAAAAGACAACAAC3′	5′AAACCAGTCAAACAAATACCAG3′
*TTN*	chr2: 179454374	5′GGTGGCTCAGAAATAACAAAC3′	5′TTAGATGAATCAGCACGGGT3′
*TTN*	chr2:179454375	5′GGTGGCTCAGAAATAACAAAC3′	5′TTAGATGAATCAGCACGGGT3′
*TTN*	chr2:179469995	5′GATGATGAAGGTGCGGAA3′	5′TTCTTAAACAGACACTGGATGC3′
*TTN*	chr2:179469996	5′GATGATGAAGGTGCGGAA3′	5′TTCTTAAACAGACACTGGATGC3′
*TTN*	chr2:179594978	5′CCCCCTTACTTTGTGGAA3′	5′AAAGTGAGGAGATGTAGAGACC3′
*TTN*	chr2:179596532	5′CTTGTGTTGCCAAAGTTATG3′	5′TCAGAAAAGCCAGTCCCT3′
*TTN*	chr2:179598095	5′TTCAGATTAGTTTTGGAGGC3′	5′AAAAGGTCAATATAGAAGAGTGC3′
*TTN*	chr2:179603055	5′TATTGTCTTCTTTTGCCTTCA3′	5′TGTTACTGTCTTGGTTGTTGG3′
*TTN*	chr2:179603066	5′TATTGTCTTCTTTTGCCTTCA3′	5′TGTTACTGTCTTGGTTGTTGG3′
*TTN*	chr2:179623776	5′AAAGACAAGAAAATCAAGCCA3′	5′CACAATGAAAAGTGGTAAAGGA3′
*TTN*	chr2:179643647	5′AGCGCATCAAACATGGAGAA3′	5′ACAGGGGCAAGAAATAAAAACT3′
*MYOZ2*	chr4: 120072176	5′AGCATCCCCAGAGACATCA3′	5′AGTACCAGACTTCCCTACACAAT3′
*MYOZ2*	chr4: 120072183	5′AGCATCCCCAGAGACATCA3′	5′AGTACCAGACTTCCCTACACAAT3′
*PDLIM3*	chr4: 186429649	5′TTTTTCACCGTCTTCCCTTT3′	5′TTTGGTCCTTACCTGATTTCAT3′
*PDLIM3*	chr4: 186429688	5′TTTTTCACCGTCTTCCCTTT3′	5′TTTGGTCCTTACCTGATTTCAT3′
*LAMA4*	chr6: 112512883	5′ATTCCTCTTCAGATGTGCTC3′	5′TGTTGTGTTTGTGTCTCCTAGT3′
*KIAA0196*	chr8: 126056890	5′ATTCCAAGTCAACACCCTA3′	5′CTTACAAAAACAGTCTAATCCTA3′

### Protein physics and chemical parameters prediction

To compare the potential functional influences of the possible risk variants on the protein's physical–chemical properties, we focused on analyzing the changes in hydrophobicity, the transmembrane domain and protein phosphorylation of amino acids induced by the mutant and wild type of *LMNA*, *LAMA4* and *RYR2* genes. ProtScale (https://web.expasy.org/protscale/) and NetPhos (http://www.cbs.dtu.dk/services/NetPhos/)were used for hydrophobicity, the transmembrane domain and phosphorylation analysis [22].

### Joint pathogenicity

According to our previous study [1], *LMNA*, *LAMA4* and *RYR2* are the important risk genes participating in cardiomyopathy pathogenesis. Additionally, these three genetic variants existed in II: 5 proband. Therefore, it was necessary to analyze these three variants' joint pathogenicity and whether the joint pathogenicity aggravates the clinical phenotype of ARVC/D. ORVAL (https://orval.ibsquare.be) is the first web bioinformatics platform to explore predicted candidate disease-causing variant combinations, aiming to aid in uncovering the causes of oligogenic diseases (i.e., diseases caused by variants in a small number of genes). This tool integrates innovative machine learning methods for combinatorial variant pathogenicity prediction, further external annotations and interactive and exploratory visualization techniques [22, 23].

## Results

### Pathogenic risk analysis of variants

In this family (Fig. 1 and Table 3), a set of candidate genes associated with cardiomyopathies and arrhythmias were screened using the WES data of II: 5. The results showed that II: 5 carried 38 variants associated with cardiomyopathy and arrhythmia, including *LMNA*, *ACTA1*, *RYR2*, *MYL2*, *MYH7*, *TPM1*, *TTN*, *MYOZ2*, *PDLIM3*, *LAMA4* and *KIAA0196*. As demonstrated in Sanger sequencing, II: 5 carried with four variants of *LMNA* p.A242V (NM_001257374, c.C389T), *LAMA4* p.A225P (NM_001282626, c.C725T), *RYR2* p.T858M (NM_001035, c.C2573T) and *KIAA0196* p.H852R (NM_014846, c.A2555G). In contrast, the other variants of *ACTA1*, *MYL2*, *MYH7*, *TPM1*, *TTN*, *MYOZ2* and *PDLIM3* were negative, termed as “false positive” detected by WES.

**Table 3:** The potential pathogenic variants of the proband

Chr	Start	Func	Gene	Amino acid change	1000 g all	SIFT	Polyphen2	MetaSVM	CADD	Clinvar	ACMG
**chr1**	**156104681**	**Exonic**	***LMNA***	**NM_001282626:exon4:c.C725T:p.A242V**	**–**	**D(0.01)**	**D(0.979)**	**D(0.9)**	**33**	**LP**	**VUS**
chr1	229568310	Exonic	*ACTA1*	NM_001100:exon3:c.G447T:p.R149S	–	D(0)	B(0.044)	D(0.656)	22.6	–	
**chr1**	**237666765**	**Exonic**	***RYR2***	**NM_001035:exon22:c.C2573T:p.T858M**	**–**	**D(0.01)**	**B(0.247)**	**D(0.723)**	**25.8**	**US**	**LB**
chr12	111356969	Exonic	*MYL2*	NM_000432:exon2:c.G32A:p.G11E	–	T(0.25)	B(0)	T(− 0.897)	9.755	–	–
chr14	23902749	Exonic	*MYH7*	NM_000257:exon3:c.T193C:p.Y65H	–	T(0.08)	B(0.001)	T(− 0.882)	0.001	–	–
chr15	63336266	Exonic	*TPM1*	NM_000366:exon2:c.G155C:p.G52A	–	T(0.73)	B(0.016)	D(0.22)	3.764	–	–
chr2	179414991	Exonic	*TTN*	NM_003319:exon165:c.T64379C:p.I21460T	0.00019968	D(0)	B(0)	T(− 1.033)	5.772	–	–
chr2	179426798	Exonic	*TTN*	NM_003319:exon154:c.T56866A:p.S18956T	–	D(0)	B(0)	T(− 1.028)	1.396	–	–
chr2	179435192	Exonic	*TTN*	NM_003319:exon154:c.A48472G:p.T16158A	–	D(0)	B(0.023)	T(− 0.872)	0.386	–	–
chr2	179435225	Exonic	*TTN*	NM_003319:exon154:c.G48439A:p.V16147I	–	D(0)	B(0.002)	T(− 0.97)	15.47	–	–
chr2	179435281	Exonic	*TTN*	NM_003319:exon154:c.G48383A:p.R16128K	–	D(0)	B(0.001)	T(− 1.017)	14.91	–	–
chr2	179435636	Exonic	*TTN*	NM_003319:exon154:c.C48028A:p.H16010N	–	D(0)	B(0)	T(− 0.929)	15.99	–	–
chr2	179435664	Exonic	*TTN*	NM_003319:exon154:c.T48000G:p.H16000Q	–	D(0)	B(0)	T(− 1.038)	2.385	–	–
chr2	179435678	Exonic	*TTN*	NM_003319:exon154:c.A47986G:p.I15996V	–	D(0)	B(0.002)	T(− 1.005)	9.277	–	–
chr2	179436004	Exonic	*TTN*	NM_003319:exon154:c.G47660A:p.S15887N	–	D(0)	B(0.019)	T(− 0.722)	17.16	–	–
chr2	179436043	Exonic	*TTN*	NM_003319:exon154:c.G47621A:p.R15874K	–	D(0)	B(0)	T(− 1.031)	13.67	–	–
chr2	179436062	Exonic	*TTN*	NM_003319:exon154:c.A47602G:p.I15868V	–	D(0)	B(0)	T(− 0.989)	8.062	–	–
chr2	179439808	Exonic	*TTN*	NM_003319:exon154:c.C43856A:p.T14619N	–	D(0)	B(0.001)	T(− 1.0)	2.982	–	–
chr2	179440715	Exonic	*TTN*	NM_003319:exon154:c.A42949G:p.N14317D	–	D(0)	B(0)	T(− 1.022)	10.88	–	–
chr2	179442904	Exonic	*TTN*	NM_003319:exon150:c.C41143A:p.L13715I	–	D(0)	B(0.001)	T(− 1.03)	13.5	–	–
chr2	179454374	Exonic	*TTN*	NM_003319:exon132:c.A34883C:p.D11628A	–	D(0)	D(0.999)	T(− 0.591)	16.01	–	–
chr2	179454375	Exonic	*TTN*	NM_003319:exon132:c.G34882A:p.D11628N	–	D(0)	D(0.999)	T(− 0.573)	21.3	–	–
chr2	179469995	Exonic	*TTN*	NM_003319:exon108:c.G26714C:p.S8905T	–	D(0)	B(0)	T(− 0.884)	11.48	–	–
chr2	179469996	Exonic	*TTN*	NM_003319:exon108:c.A26713G:p.S8905G	–	D(0)	B(0.014)	T(− 1.001)	7.502	–	–
chr2	179594978	Exonic	*TTN*	NM_133378:exon59:c.A14417G:p.K4806R	–	D(0)	P(0.489)	T(− 0.5)	14.71	–	–
chr2	179596532	Exonic	*TTN*	NM_133378:exon55:c.T13338G:p.H4446Q	–	D(0)	B(0)	T(− 1.023)	0.001	–	–
chr2	179598095	Exonic	*TTN*	NM_133378:exon51:c.A12193T:p.I4065L	–	D(0)	B(0.001)	T(− 0.979)	12.51	–	–
chr2	179603055	Exonic	*TTN*	NM_003319:exon46:c.C13036G:p.L4346V	–	D(0)	P(0.652)	T(− 0.883)	10.8	–	–
chr2	179603066	Exonic	*TTN*	NM_003319:exon46:c.A13025G:p.K4342R	–	D(0)	B(0.002)	T(− 0.88)	14.9	–	–
chr2	179623776	Exonic	*TTN*	NM_003319:exon43:c.C10100T:p.T3367I	–	D(0)	B(0.004)	T(− 0.824)	12.56	–	–
chr2	179643647	Exonic	*TTN*	NM_003319:exon23:c.G4024A:p.G1342R	–	D(0)	B(0.316)	T(− 0.91)	9.399	–	–
chr4	120072176	Exonic	*MYOZ2*	NM_016599:exon3:c.C226G:p.Q76E	–	T(1)	B(0.001)	T(− 1.009)	0.31	–	–
chr4	120072183	Exonic	*MYOZ2*	NM_016599:exon3:c.G233A:p.R78K	–	T(1)	B(0.001)	T(− 0.98)	10.29	–	–
chr4	186429649	Exonic	*PDLIM3*	NM_014476:exon5:c.G466C:p.V156L	–	T(0.65)	B(0)	T(− 0.937)	6.766	–	–
chr4	186429688	Exonic	*PDLIM3*	NM_014476:exon5:c.T427G:p.C143G	–	T(0.38)	B(0)	T(− 1.084)	1.276	–	–
**chr6**	**112512883**	**Exonic**	***LAMA4***	**NM_001105206:exon6:c.G673C:p.A225P**	**–**	**D(0.01)**	**D(1)**	**D(0.052)**	**27.4**	**LB**	**B**
**chr8**	**126056890**	**Exonic**	***KIAA0196***	**NM_014846:exon21:c.A2555G:p.H852R**	**–**	**T(0.43)**	**B(0.006)**	**T(0.94)**	**9.419**	**–**	**VUS**

Bold indicates the variants positively carried by the family members, varified by the Sanger sequencing

Chr: chromosome. Fre: frequency. Het: heterozygosis. Hom: homozygosis. GnomAD: frequency of the existing variant in gnomAD exomes combined population. Local Fre: frequency information about this SNP in sequencing samples of over 200 normal people collected locally. Local frequency: 0–0.01 = A; 0.01–0.05 is B (including 0.01 and 0.05); 0.05–1 is C. P: possibly damaging; T: tolerated; U: unknown. 1000G: 1000 Genomes Project databases (2014version). B: benign. D: deleterious. US: uncertain significance. LB: likely benign. LP: likely pathogenic. –: no report

The variants of *LMNA* p.A242V and *LAMA4* p.A225P are predicated as “deleterious” by SIFT, Polyphen2, and MetaSVM algorithms; both of the CADD predicting scores are 33 and 27.4, respectively. According to the Clinvar database, the *LMNA* p.A242V (rs ID: rs397517906, gnomAD: 0.00001) has been reported in the literature in one individual affected with ARVC/D and individuals affected with DCM. This variant was also identified in three Caucasian adults (one with ARVC/D, one with clinical features of ARVC/D, and one with DCM) and segregated with disease (DCM, unspecified cardiomyopathy, chronic HF, sudden cardiac death) in six affected relatives from two families (including three obligate carriers). *LMNA* p.A242V was also found in the DCM family. Therefore, based on the evidence, *LMNA* p.A242V is classified as “LP” in the Clinvar database [https://www.ncbi.nlm.nih.gov/clinvar/variation/VCV000048076.13]. However, according to ACMG guidelines, the pathogenic risk of *LMNA* p.A242V is classified as “VUS”.

In contrast, the pathogenic risk of *LAMA4* p.A225P (rs ID: rs782121531, gnomAD: 0.00006) is classified as “LB” and “B” by the Clinvar database and ACMG guidelines but lacked genetic verification in familial/sporadic cases of cardiovascular diseases (https://www.ncbi.nlm.nih.gov/clinvar/variation/VCV000213580.6). The variant of *RYR2* p.T858M (rs ID: rs377068202, gnomAD: 0.00004) with the CADD predicting score of 25.8 is predicted as “deleterious” by the SIFT and MetaSVM algorithms but “tolerated” by the Polyphen2 algorithm. It is classified as “US” and “LB” by the Clinvar database (https://www.ncbi.nlm.nih.gov/clinvar/variation/VCV000532370.5) and ACMG guidelines due to this variant not being reported in individuals with cardiovascular disorders. The variant of *KIAA0196* p.H852R [rs ID: not reported, gnomAD: not reported, according to Exome Variant Server (NHLBI Exome Sequencing Project, ESP)] with the CADD predicting score of 9.42 is predicated as “tolerated” by these three algorisms and classified as “VUS” by ACMG guidelines.

### Conservation analysis

**Fig. 4:**
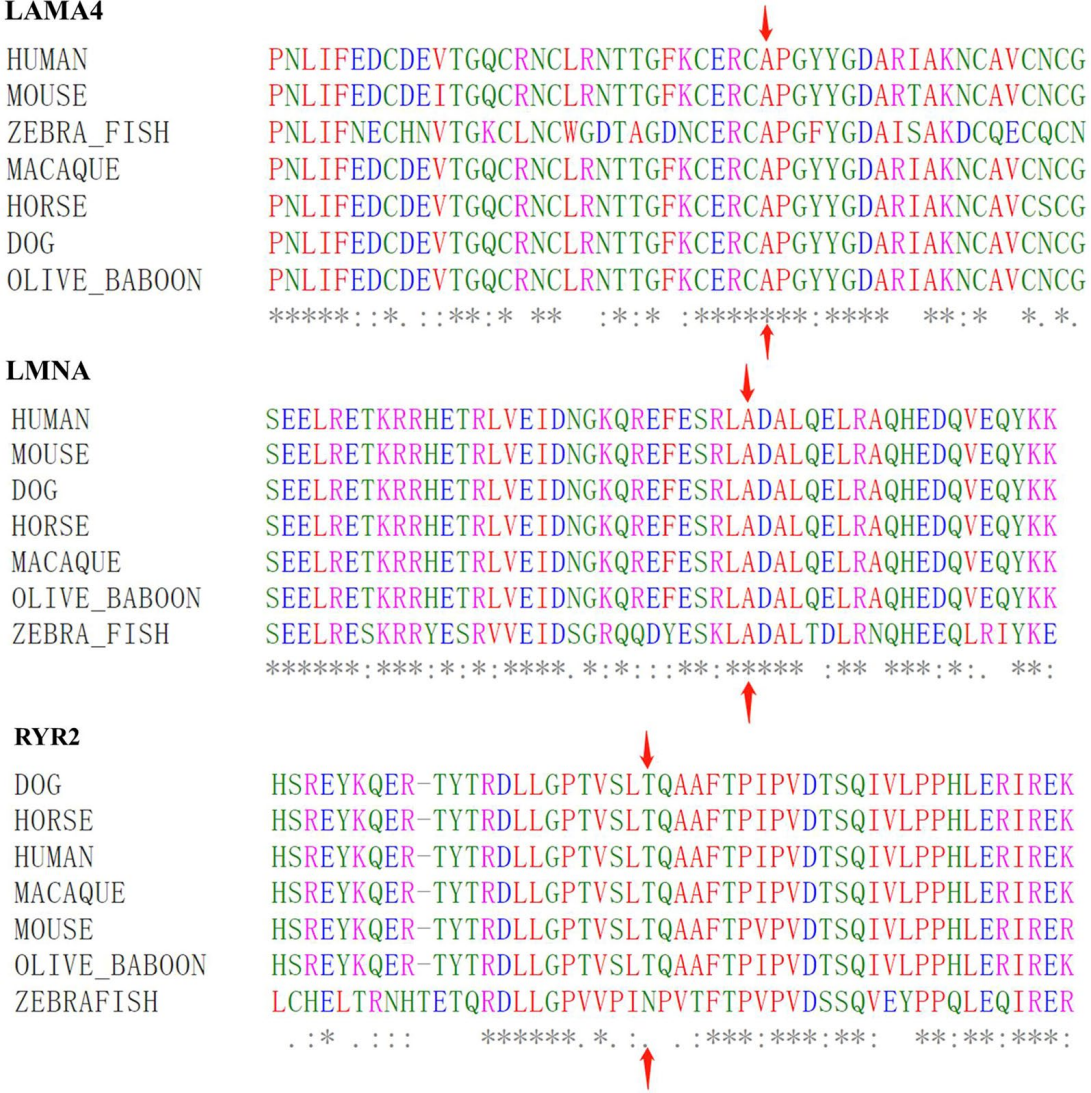
The conservative analysis of the risk genetic variants

The conservation analyses (Fig. 4) demonstrate that amino acid sequences are highly conserved in the mutant sites (*LMNA* p.A242V and *LAMA4* p.A225) among the species, including humans, mice, dogs, horses, macaque, olive baboon and zebrafish. The mutant site of *RYR2* p.T858 is highly conserved among the species, including humans, mice, dogs, horses, macaque, and olive baboons, but not in the zebrafish.

### Hydrophobicity and phosphorylation prediction

The hydrophobicity analysis results (Table 4) show that the *LMNA* p.A242V, *LAMA4* p.A225P and *RYR2* p.T858M increase the hydrophobicity of their corresponding amino acid residues and their nearby sequences by 0.266, 0.378 and 0.289, respectively. The three variant sites are located in the non-transmembrane region. *LMNA* p.A242V does not affect the phosphorylation modification of its corresponding amino acid and nearby amino acid sequences. The *LAMA4* p.A225P and *RYR2* p.T858M lead to changes in protein phosphorylation at or near their corresponding amino acid sites. The *LAMA4* p.A225P results in the shift of p.229Y amino acid from non-phosphorylation to phosphorylation. The *RYR2* p.T858M results in the disappearance of its corresponding amino acid phosphorylation modification.

**Table 4:** The changes of hydrophobicity and phosphorylation induced by LAMA4, LMNA and RYR2 variants

Location		219	220	221	222	223	224	225	226	227	228	229	230	231
*LAMA4:NM_001105206:exon6:c.G673C:p.A225P*														
Amino acid	W	F	K	C	E	R	C	**A**	P	G	Y	Y	G	D
	M	F	K	C	E	R	C	**P**	P	G	Y	Y	G	D
Hydrophobicity	W	− 1.322	− 0.656	− 0.38	− 0.48	− 0.48	− 0.93	**− 0.64**	− 0.97	− 0.97	− 0.27	− 1.04	− 0.74	− 0.37
	M	− 1.322	− 0.656	− 0.76*	− 0.86*	− 0.86*	− 1.31*	− 1.02*	− 1.34*	− 1.34*	− 0.64*	− 1.42*	− 0.74	− 0.37
Transmembrane	W	Outside	Outside	Outside	Outside	Outside	Outside	**Outside**	Outside	Outside	Outside	Outside	Outside	Outside
	M	Outside	Outside	Outside	Outside	Outside	Outside	**Outside**	Outside	Outside	Outside	Outside	Outside	Outside
Phosphorylation	W	–	–	–	–	–	–	–	–	–	–	No	–	–
	M	–	–	–	–	–	–	–	–	–	–	Yes	–	–

Location		236	237	238	239	240	241	242	243	244	245	246	247	248
*LMNA: NM_001282626:exon4:c.C725T:p.A242V*														
Amino acid	W	E	F	E	S	R	L	**A**	D	A	L	Q	E	L
	M	E	F	E	S	R	L	**V**	D	A	L	Q	E	L
Hydrophobicity	W	− 2.422	− 1.956	− 1.32	− 1.32	− 0.62	0.19	**− 0.51**	− 0.51	− 0.00	− 0.00	− 0.22	− 0.811	− 0.778
	M	− 2.422	− 1.956	− 1.06*	− 1.06*	− 0.36*	0.46*	− 0.24*	− 0.24*	0.27*	0.27*	0.04*	− 0.811	− 0.778
Transmembrane	W	Outside	Outside	Outside	Outside	Outside	Outside	**Outside**	Outside	Outside	Outside	Outside	Outside	Outside
	M	Outside	Outside	Outside	Outside	Outside	Outside	**Outside**	Outside	Outside	Outside	Outside	Outside	Outside
Phosphorylation	W	–	–	–	–	–	–	–	–	–	–	–	–	–
	M	–	–	–	–	–	–	–	–	–	–	–	–	–

Location		852	853	854	855	856	857	858	859	860	861	862	863	864
*RYR2: NM_001035:exon22:c.C2573T:p.T858M*														
Amino acid	W	G	P	T	V	S	L	**T**	Q	A	A	F	T	P
	M	G	P	T	V	S	L	**M**	Q	A	A	F	T	P
Hydrophobicity	W	0.033	0.956	1.27	0.46	0.23	0.48	**0.97**	0.97	0.32	0.91	0.31	0.856	0.856
	M	0.033	0.956	1.56*	0.74*	0.52*	0.77*	1.26*	1.26*	0.61*	1.20*	0.60*	0.856	0.856
Transmembrane	W	Outside	Outside	Outside	Outside	Outside	Outside	**Outside**	Outside	Outside	Outside	Outside	Outside	Outside
	M	Outside	Outside	Outside	Outside	Outside	Outside	**Outside**	Outside	Outside	Outside	Outside	Outside	Outside
Phosphorylation	W	–	–	Yes	–	–	–	**Yes**	–	–	–	–	Yes	–
	M	–	–	Yes	–	–	–	**No**	–	–	–	–	Yes	–

Bold indicates the amino acid substitutions and their changes in physical and chemical properties, induced by the variants of LAMA4 p.A225P, LMNA p.A242V and RYR2 p.T858M in this family

W, wild-type; M, mutation, *, a significant difference; –, not reported

### Join pathogenicity

We analyzed the joint pathogenicity of potentially candidate pathogenic sites, including the variants of *LMNA* p.A242V, *LAMA4* p.A225P and *RYR2* p.T858M. The value of support score (SS)/classification score (CS) of joint pathogenicity reached 100.00/0.95 (99% zone candidate disease-causing) for *LMNA* p.A242V/*LAMA4* p.A225P, 100.00/0.88 (99% zone candidate disease-causing) for *LAMA4* p.A225P/*RYR2* p.T858M and 98.60/0.75 (95% zone candidate disease-causing) for *LMNA* p.A242V/*RYR2* p.T858M, which indicates that there are the high risks of joint pathogenicity for cardiomyopathy among these three variants.

### Cosegregation analysis

Due to III: 1 with early ECG abnormality and II: 5 proband with ARVC/D and complex complications carrying *LMNA* p.A242V and *LMNA* p.A242V/*LAMA4* p.A225P (Fig. 1 and Table 5), respectively, we speculate that the patients of I: 1 and II: 1 who died of RVHF and cerebral thromboembolism also carry *LMNA* p.A242V. Additionally, the *LAMA4* p.A225P was inherited from I: 2 without ARVC/D or ECG abnormality. These findings indicated that *LMNA* p.A242V might be an important risk factor for ARVC/D, ECG abnormality and cerebral thromboembolism.

**Table 5:** The variant distribution in the familial members

Gene	Amino acid Change	I:2	II:3	II:4	II:5	III:1	III:2	III:3	III:4	III:5	III:6	III:7	III:8
***LMNA***	**NM_001282626:exon4:c.C725T:p.A242V**	−/−	−/−	−/−	**−/+**	−/+	−/−	−/−	−/−	−/−	**−/−**	−/−	−/−
*ACTA1*	NM_001100:exon3:c.G447T:p.R149S	−/−	−/−	−/−	−/−	−/−	−/−	−/−	−/−	−/−	−/−	−/−	−/−
***RYR2***	**NM_001035:exon22:c.C2573T:p.T858M**	**−/+**	−/−	**−/+**	**−/+**	−/−	−/−	−/−	−/−	−/−	−/+	−/+	−/−
*MYL2*	NM_000432:exon2:c.G32A:p.G11E	−/−	−/−	−/−	−/−	−/−	−/−	−/−	−/−	−/−	−/−	−/−	−/−
*MYH7*	NM_000257:exon3:c.T193C:p.Y65H	−/−	−/−	−/−	−/−	−/−	−/−	−/−	−/−	−/−	−/−	−/−	−/−
*TPM1*	NM_000366:exon2:c.G155C:p.G52A	−/−	−/−	−/−	−/−	−/−	−/−	−/−	−/−	−/−	−/−	−/−	−/−
*TTN*	NM_003319:exon165:c.T64379C:p.I21460T	−/−	−/−	−/−	−/−	−/−	−/−	−/−	−/−	−/−	−/−	−/−	−/−
*TTN*	NM_003319:exon154:c.T56866A:p.S18956T	−/−	−/−	−/−	−/−	−/−	−/−	−/−	−/−	−/−	−/−	−/−	−/−
*TTN*	NM_003319:exon154:c.A48472G:p.T16158A	−/−	−/−	−/−	−/−	−/−	−/−	−/−	−/−	−/−	−/−	−/−	−/−
*TTN*	NM_003319:exon154:c.G48439A:p.V16147I	−/−	−/−	−/−	−/−	−/−	−/−	−/−	−/−	−/−	−/−	−/−	−/−
*TTN*	NM_003319:exon154:c.G48383A:p.R16128K	−/−	−/−	−/−	−/−	−/−	−/−	−/−	−/−	−/−	−/−	−/−	−/−
*TTN*	NM_003319:exon154:c.C48028A:p.H16010N	−/−	−/−	−/−	−/−	−/−	−/−	−/−	−/−	−/−	−/−	−/−	−/−
*TTN*	NM_003319:exon154:c.T48000G:p.H16000Q	−/−	−/−	−/−	−/−	−/−	−/−	−/−	−/−	−/−	−/−	−/−	−/−
*TTN*	NM_003319:exon154:c.A47986G:p.I15996V	−/−	−/−	−/−	−/−	−/−	−/−	−/−	−/−	−/−	−/−	−/−	−/−
*TTN*	NM_003319:exon154:c.G47660A:p.S15887N	−/−	−/−	−/−	−/−	−/−	−/−	−/−	−/−	−/−	−/−	−/−	−/−
*TTN*	NM_003319:exon154:c.G47621A:p.R15874K	−/−	−/−	−/−	−/−	−/−	−/−	−/−	−/−	−/−	−/−	−/−	−/−
*TTN*	NM_003319:exon154:c.A47602G:p.I15868V	−/−	−/−	−/−	−/−	−/−	−/−	−/−	−/−	−/−	−/−	−/−	−/−
*TTN*	NM_003319:exon154:c.C43856A:p.T14619N	−/−	−/−	−/−	−/−	−/−	−/−	−/−	−/−	−/−	−/−	−/−	−/−
*TTN*	NM_003319:exon154:c.A42949G:p.N14317D	−/−	−/−	−/−	−/−	−/−	−/−	−/−	−/−	−/−	−/−	−/−	−/−
*TTN*	NM_003319:exon150:c.C41143A:p.L13715I	−/−	−/−	−/−	−/−	−/−	−/−	−/−	−/−	−/−	−/−	−/−	−/−
*TTN*	NM_003319:exon132:c.A34883C:p.D11628A	−/−	−/−	−/−	−/−	−/−	−/−	−/−	−/−	−/−	−/−	−/−	−/−
*TTN*	NM_003319:exon132:c.G34882A:p.D11628N	−/−	−/−	−/−	−/−	−/−	−/−	−/−	−/−	−/−	−/−	−/−	−/−
*TTN*	NM_003319:exon108:c.G26714C:p.S8905T	−/−	−/−	−/−	−/−	−/−	−/−	−/−	−/−	−/−	−/−	−/−	−/−
*TTN*	NM_003319:exon108:c.A26713G:p.S8905G	−/−	−/−	−/−	−/−	−/−	−/−	−/−	−/−	−/−	−/−	−/−	−/−
*TTN*	NM_133378:exon59:c.A14417G:p.K4806R	−/−	−/−	−/−	−/−	−/−	−/−	−/−	−/−	−/−	−/−	−/−	−/−
*TTN*	NM_133378:exon55:c.T13338G:p.H4446Q	−/−	−/−	−/−	−/−	−/−	−/−	−/−	−/−	−/−	−/−	−/−	−/−
*TTN*	NM_133378:exon51:c.A12193T:p.I4065L	−/−	−/−	−/−	−/−	−/−	−/−	−/−	−/−	−/−	−/−	−/−	−/−
*TTN*	NM_003319:exon46:c.C13036G:p.L4346V	−/−	−/−	−/−	−/−	−/−	−/−	−/−	−/−	−/−	−/−	−/−	−/−
*TTN*	NM_003319:exon46:c.A13025G:p.K4342R	−/−	−/−	−/−	−/−	−/−	−/−	−/−	−/−	−/−	−/−	−/−	−/−
*TTN*	NM_003319:exon43:c.C10100T:p.T3367I	−/−	−/−	−/−	−/−	−/−	−/−	−/−	−/−	−/−	−/−	−/−	−/−
*TTN*	NM_003319:exon23:c.G4024A:p.G1342R	−/−	−/−	−/−	−/−	−/−	−/−	−/−	−/−	−/−	−/−	−/−	−/−
*MYOZ2*	NM_016599:exon3:c.C226G:p.Q76E	−/−	−/−	−/−	−/−	−/−	−/−	−/−	−/−	−/−	−/−	−/−	−/−
*MYOZ2*	NM_016599:exon3:c.G233A:p.R78K	−/−	−/−	−/−	−/−	−/−	−/−	−/−	−/−	−/−	−/−	−/−	−/−
*PDLIM3*	NM_014476:exon5:c.G466C:p.V156L	−/−	−/−	−/−	−/−	−/−	−/−	−/−	−/−	−/−	−/−	−/−	−/−
*PDLIM3*	NM_014476:exon5:c.T427G:p.C143G	−/−	−/−	−/−	−/−	−/−	−/−	−/−	−/−	−/−	−/−	−/−	−/−
***LAMA4***	**NM_001105206:exon6:c.G673C:p.A225P**	**−/+**	**−/+**	**−/+**	**−/+**	−/−	−/−	−/−	−/−	**−/+**	**−/+**	−/−	**−/+**
***KIAA0196***	**NM_014846:exon21:c.A2555G:p.H852R**	−/−	−/+	−/−	**−/+**	−/−	−/−	**−/+**	**−/+**	−/−	−/−	−/−	**−/+**

Bold indicates the variants positively carried by the family members, varified by the Sanger sequencing

−/−, not carried. −/+, heterozygously carried

The II: 3 with ARVC/D carry *LAMA4* p.A225P (negative of *LMNA* p.A242V and *RYR2* p.T858M), while the II: 4 and III: 6 with hereditary ECG abnormality carry *LAMA4* p.A225P/*RYR2* p.T858M, both of which inherited from I: 2, suggesting that *LAMA4* p.A225P may be a pathogenic etiology of ARVC/D and hereditary ECG abnormality. The III: 7 carrying *RYR2* p.T858M showed normal ECG and no ARVC/D manifestation. Therefore, we can’t determine whether *RYR2* p.T858M contributes to hereditary ECG abnormality for II: 4 and III: 6 and ARVC/D for II: 5. The III: 1 with early ECG abnormality and II: 3 with ARVC/D didn’t carry *RYR2* p.T858M, suggesting that the *RYR2* p.T858 may not be the necessary reason for ARVC/D and ECG abnormality. For II: 2 patient, malignant ventricular arrhythmia and thromboembolism resulting from ARVC/D would lead to sudden death. Because of a lack of blood DNA samples before II: 2 death, clinical/genetic information and the possibility of incomplete penetrance, we can’t evaluate whether the *RYR2* p.T858M was associated with unexplained sudden death at night.

## Discussion

In this study, we first demonstrated that *LMNA* might participate in the pathogenesis of familial ARVC/D with RVHF and cerebral thromboembolism, while *LAMA4* p.A225P may be associated with ARVC/D and hereditary ECG abnormality. The patient carrying *LMNA* p.A242V/*LAMA4* p.A225 presented with ARVC/D and complex complications, including RVHF, atrial fibrillation, atrial standstill, multifocal ventricular premature, right bundle block and third-degree atrioventricular block, which were the high-risk factors for thromboembolism and sudden death.

### *LMNA* p.A242V may be associated with ARVC/D and thromboembolism

*LMNA*, located on human chromosome 1q21.2–21.3, encodes nuclear Lamin A/C, intermediate filament proteins that are components of the nuclear laminas. The nuclear lamina is attached to the inner nuclear membrane by the binding of lamins to integral proteins of the membrane, which therefore not only functions as the structural support and mechanotransduction to the nucleus but also participates in the chromatin organization, splicing factor compartment organization, DNA replication, transcription regulation (modifying the RNA polymerase II transcription), DNA repair, posttranslational modification, signal transduction pathways and nucleocytoskeletal connections [24–28].

*LMNA* mutations abnormally increase the signaling by extracellular signal-regulated kinase 1 and kinase 2 (ERK1/2), Jun N-terminal kinase (JNK) and mitogen-activated protein kinases (MAPK), protein kinase B/mammalian target of rapamycin complex 1 and transforming growth factor-β [29–31]. *LMNA* mutations lead to a loss of nuclear stability, enhanced sensitivity to mechanical strain, drastically enhanced nuclear envelope fragility, apoptosis, endoplasmic reticulum stress, abnormal calcium handling of endoplasmic reticulum, connexin 43 downregulation and abnormal calcium oscillations upon different cellular stresses, such as hypertonic, hypoxic, oxidative stresses and mechanical stress [19, 32–39]. The inherited disorders related to *LMNA* mutations include muscular dystrophies, cardiac conduction defects, and cardiomyopathy (such as DCM and ARVC/D). The latter is characterized by progressive HF, complicated by life-threatening arrhythmias, and eventually death or heart transplantation [24, 40–49]. For the carriers with *LMNA* mutations, cardiac dysrhythmias account for 92% of patients after the age of 30-year old. HF was reported in 64% after the age of 50. Sudden death was the most frequently reported mode of death (46%) in cardiac and neuromuscular phenotypes. Carriers with *LMNA* mutations often received a pacemaker (28%) [50]. Moreover, there is a possible link between *LMNA* mutation and arterial/venous thromboembolism [40]. In our study, the *LMNA* p.A242V increases the hydrophobicity of its corresponding amino acid residues and their nearby sequences, which may affect the function of the LMNA protein. More interestingly, based on the cosegregation of clinical phenotypes and genotypes, our important finding first demonstrated that *LMNA* p.A242V might be associated with aberrant ARVC/D, severe low-limb edema induced by RVHF and thromboembolism, especially cerebral thromboembolism. Therefore, anticoagulant therapy may need to be considered for patients with ARVC/D caused by *LMNA* p.A242V.

### *LAMA4* p.A225P may be associated with ARVC/D and hereditary ECG abnormality

Laminins are important glycoproteins for the basement membrane extracellular matrix that provide cells with structural stability and signaling functions. *LAMA4*, the gene encoding the laminin α4 chain, is developmentally regulated during embryogenesis and localized mainly in the basement membranes of blood vessels of the adult heart and the peripheral sarcolemma of cardiomyocytes. The LAMA4 interacted with integrin molecules (especially α3ß1 integrins) and its connection with ILK. By controlling AKT kinase activity and its connection to the cytoskeleton via Parvin, the LAMA4-integrin-ILK pathway is central in converting extracellular signals into intracellular survival pathways [51].

*LAMA4* knockdown in zebrafish causes defects in endothelial cells, cardiac dysfunction, and hemorrhage in embryos. *LAMA4*^*−/−*^ mice showed microcirculation dysfunction, characterized by irregularity of capillaries and enlargement of space between cardiomyocyte and its adjacent capillary vessels, which might cause insufficient oxygen supply and cardiac phenotypes including cardiac dysfunction, cardiomyocyte degeneration and subsequent compensatory hypertrophy [52]. According to previous reports, *LAMA4* p.P943L (c.2828C > T) and p.R1073X (c.3217C > T) mutations have significantly reduced the extracellular matrix in cardiomyocytes, which are associated with DCM and HCM [51]. The digenic mutations of *LAMA4* p.D1309N (NM_001105206.2, c.3925G > A) and *MYH7* (part of the sarcomere) p.E924K (NM_000257.3, c.2770G > A) induced infantile DCM [53]. In our study, *LAMA4* p.A225P predicated as “deleterious,” increases the hydrophobicity of its corresponding amino acid residues and their nearby sequences and changes the 229^th^ amino acid from non-phosphorylation to phosphorylation. According to the cosegregation of clinical phenotypes and genotypes*, LAMA4* p.A225P may be an important pathogenic risk factor for ARVC/D and hereditary ECG abnormality.

### *RYR2* p.T858M is not the necessary reason for ARVC/D

The cardiac ryanodine receptor (RYR2) located in the sarcoplasmic reticulum (SR) membrane functions as a Ca^2+^ release channel characterized by Ca^2+^-induced Ca^2+^ release. The Ca^2+^ flows from the lumen of SR into the cytoplasm and activates cardiomyocyte contraction. The cardiac RYR2 protein is differentially expressed across the cardiac walls of the right ventricle, left ventricle and interventricular septum in a normal heart, with the lowest concentration expressed in the right ventricle. The RYR2 expression is reduced in all chambers in the ARVC/D canine model [54]. According to our previous study, *RYR2* mutation is common in cardiac diseases, such as catecholaminergic polymorphic ventricular tachycardia, atrial fibrillation and ARVC/D, which may cause life-threatening ventricular arrhythmias and unexplained sudden death [21]. A systematic screening of the whole coding region of *RYR2* in a large ARVC/D cohort without mutation in desmosome genes shows that putative *RYR2* mutations are frequent (9% of ARVC/D probands) and associated with ARVC/D [55].

The pathogenic mutations (Genbank accession: No. X98330. c.5654G > A, p.G1885E and c.5656G > A, p.G1886S) of *RYR2* were reported to change the subunit composition and alter the behavior of the tetramer channel complex of the RYR2 channel, which therefore causes sarcoplasmic reticulum-Ca^2+^ depletion and induces ARVC/D and HF [56]. A further study illustrates that the store-overload-induced calcium release activity is nearly completely abolished when mutations of p.G1885E and G1886S were introduced in the same RYR2 subunit. However, the Ca^2+^ loading of the intracellular stores is markedly enhanced, and the channel still displays substantial Ca^2+^ release on stimulation by five mM caffeine [57]. According to the cosegregation in our study, *RYR2* p.T858M may not be the necessary reason for ARVC/D and ECG abnormality. Due to a lack of blood DNA samples and detailed clinical information on I: 1, II: 1 and II: 2 before death, we can’t evaluate the influence of *RYR2* p.T858M on the risk of ARVC/D, thromboembolism, ECG abnormality and sudden death.

### Limitation

We can’t determine the patient's genotypes of I: 1, II: 1 and II: 2 with identical phenotypes (low-limb edema induced by RVHF) due to a lack of blood DNA and detailed clinical information before death. Meanwhile, the contribution of the *RYR2* variant to the risk of ARVC/D, thromboembolism, ECG abnormality and sudden death in this family cannot be determined. The joint pathogenic risks and their pathogenicity of *LMNA*, *LAMA4* and *RYR2* variants need to be further confirmed by other central data, cellular and animal experimental studies.

## Conclusion

Our study first illustrated that *LMNA* p.A242V might participate in the pathogenesis of familial ARVC/D with RVHF and cerebral thromboembolism, while *LAMA4* p.A225P may be associated with ARVC/D and hereditary ECG abnormality. Anticoagulant therapy may need to be considered for patients with ARVC/D induced by *LMNA* p.A242V.

## Data Availability

The data used in this study is not publicly available, but it might be available from the corresponding author upon reasonable request and permission from relevant Chinese Authorities.

## Abbreviations

ARVC/D: Arrhythmogenic right ventricular cardiomyopathy/dysplasia
HF: Heart failure
RVHF: Right ventricular heart failure
SCD: Sudden cardiac death
ICD: Implantable cardioverter-defibrillator
WES: Whole-exome sequencing
ECG: Electrocardiogram
CMR: Cardiovascular magnetic resonance
SNPs: Single-nucleotide polymorphisms
US/VUS: Uncertain significance
LB: Likely benign
B: Benign
RYR2: Ryanodine receptor-2
LMNA: Phospholamban lamin A/C
LAMA4: Laminin subunit alpha 4
